# Profiles of caregiver burden among adolescents with non-suicidal self-injury: a latent profile analysis

**DOI:** 10.3389/fpsyt.2025.1581640

**Published:** 2025-05-19

**Authors:** Yuan Qin, Jiao Liu, Jiming Duan, Bo Yang

**Affiliations:** ^1^ Department of Psychiatry, Chongqing Mental Health Center, Chongqing, China; ^2^ School of Nursing, Zunyi Medical University, Zunyi, China; ^3^ Department of Nursing, Chengdu Fifth People’s Hospital, Chengdu, China; ^4^ Department of Nursing, Chongqing Mental Health Center, Chongqing, China

**Keywords:** non-suicidal self-injury, adolescents, caregivers, caregiver burden, latent profile analysis

## Abstract

**Background:**

Non-suicidal self-injury (NSSI) in adolescents represents a significant global public health challenge, with lifetime prevalence rates demonstrating a consistent upward trajectory. Current evidence indicates that NSSI conveys elevated risks for both physical complications and psychological comorbidities, while simultaneously imposing a substantial burden on primary caregivers.

**Objective:**

This study applied latent profile analysis (LPA) to classify distinct caregiver burden profiles among adolescents with NSSI, identify modifiable determinants, and establish an empirical foundation for developing precision interventions stratified by burden type.

**Method:**

From August 2022 to May 2023, 385 caregivers of adolescents with NSSI in Sichuan Province, China, were selected as respondents and systematically assessed using six validated instruments: General Information Questionnaire, Family Burden Scale of Disease, Social Support Rating Scale, Generalized Anxiety Disorder 7-Item Scale, Patient Health Questionnaire-9, and Internalized Stigma of Mental Illness Scale. LPA with maximum likelihood estimation identified distinct caregiver burden profiles, and multinomial logistic regression to determine predictors of profile membership.

**Results:**

The caregiver burden among adolescents with NSSI can be identified into three profiles: low burden-family vulnerability profile (34.5%), moderate burden-mental health priority profile (36.6%), and high burden-economic preponderance profile (28.9%). Female caregivers (*OR* = 3.760, *p* = 0.005), rural residents (*OR*  =  3.666, *p*  =  0.027), diminished social support (*OR*  =  0.884, *p*  =  0.016), and elevated anxiety severity (*OR*  = 1.183, *p* = 0.027) exhibited heightened vulnerability to the moderate burden-mental health priority profile. Heightened depression symptoms (*OR* = 1.130, *p* = 0.037) and stronger illness-related stigma (*OR* = 1.063, *p =* 0.001) were disproportionately represented in both moderate burden-mental health priority profile and high burden-economic preponderance profile.

**Conclusions:**

This study delineates three distinct caregiver burden profiles among adolescents with NSSI, alongside sociodemographic and clinical predictors of profile membership. Tailored interventions, such as rapid mental health service linkage for high-burden subgroups and family-centered psychoeducation, can mitigate these burdens through stratified support mechanisms.

## 1 Introduction

Adolescence is a critical developmental window of heightened neuroplasticity and psychopathological vulnerability. Non-suicidal self-injury (NSSI) is a prevalent mental health concern within this population, operationally defined as the deliberate, self-inflicted destruction of bodily tissue without suicidal intent (1). Clinical presentations typically involve cutaneous cutting, severe scratching, and intentional burning (2, 3). Global data reveal a rising prevalence trend, with meta-analytic estimates indicating a 22.0% lifetime incidence in community adolescent cohorts (4). Notably, a Chinese epidemiological study demonstrated elevated rates, with 24.7% of adolescents reporting at least one NSSI episode within the preceding 12-month period (5). The behavior frequently exhibits chronicity and behavioral reinforcement patterns, with a longitudinal investigation identifying addictive characteristics in 22.1% of recurrent cases (6). Moreover, the clinical gravity of NSSI extends beyond its diagnostic categorization, functioning both as a transdiagnostic marker of psychological distress and the strongest identifiable predictor of subsequent suicidality (7, 8). Meta-analytic evidence indicates that over 50% of adolescents with NSSI exhibit clinically elevated suicide risk profiles (9). These converging lines of evidence position NSSI as an urgent global public health.

NSSI constitutes a significant threat to both physical and psychological well-being in affected individuals, while simultaneously imposing multidimensional burdens on familial systems. The protracted treatment requirements for adolescents with NSSI frequently result in substantial disruptions to caregivers’ occupational functioning, daily routines, and long-term life planning (10). Prolonged caregiving strains both physical and mental health, lowering caregivers’ quality of life and disrupting family well-being (11). This chain of effects creates a severe caregiver burden, marked by economic instability and long-term health impacts.

Caregiver burden refers to the challenges or adverse effects of the patient’s illness on the caregiver and their family, encompassing physical, psychological, emotional, interpersonal, and financial domains (12). A cross-sectional survey revealed that 25.2% of caregivers of adolescents with NSSI reported moderate-to-severe or severe burden (13). Epidemiological evidence shows elevated burden levels correlate strongly with poorer psychosocial outcomes (14). Chronic caregiving stress directly impairs care quality, undermining treatment efficacy through caregiver burnout. Furthermore, caregiver burden transcends clinical impacts to disrupt fundamental life domains, including educational pursuits, occupational performance, recreational activities, and daily functioning for patients and families (15). Crucially, this burden undermines family functioning through three pathways: impaired collaborative problem-solving, weakened collective resource management, and disrupted social support mobilization (16). In addition, some caregiver-related factors, such as anxiety (17), depression (18), low social support (19), and stigma (20), were associated with higher levels of caregiver burden.

Current research on caregiver burden in adolescents with NSSI remains notably limited. Existing studies primarily employ standardized scales to assess caregiver burden, potentially neglecting clinically meaningful heterogeneity among individuals (21). Latent profile analysis (LPA), a person-centered statistical approach, addresses this limitation by identifying latent subgroups through continuous indicator variables (22). This methodology classifies study populations into distinct profiles based on observed variable patterns, enabling systematic comparison of inter-group differences in key outcomes and characteristic identification (23). Applied to caregiver burden in NSSI adolescents, LPA facilitates: (a) identification of distinct burden phenotypes, (b) estimation of subtype prevalence rates, and (c) demographic profiling across profiles. Notably, no prior studies have implemented LPA to investigate caregiver burden among Chinese adolescents with NSSI. This study first applies LPA to identify burden profiles in this population and analyze associated predictors. This finding aims to offer clinicians an evidence-based framework for caregiver burden stratification, targeted intervention design, and personalized support delivery, effectively alleviating caregiver distress.

## 2 Methods

### 2.1 Participants

A total of 385 questionnaires were distributed in this study, with 374 valid responses retained, yielding an effective response rate of 98.9%. Inclusion criteria comprised (1) adolescents with clinician-confirmed Diagnostic and Statistical Manual of Mental Disorders, Fifth Edition (DSM-5) NSSI; (2) adolescents aged 13–18 years; and (3) participants who are the primary caregivers, legal guardians, or direct financial sources for the patients. The exclusion criteria comprised (1) adolescents with severe somatic disorders (which may introduce unrelated pathophysiological factors affecting self-injury assessment); (2) caregivers with severe health conditions (which could compromise caregiving capacity and introduce reporting biases); and (3) those experiencing major traumatic events unrelated to the adolescents’ condition (to isolate NSSI-specific triggers from external stressors), thereby ensuring homogeneity in evaluating caregiver burden associated with adolescent NSSI.

### 2.2 Procedures

Participants comprised caregivers of adolescent inpatients with NSSI recruited from a psychiatric care setting. Data collection occurred via secured electronic questionnaires administered on discharge day following standardized protocols. Prior to study enrollment, prospective participants were provided with a two-day deliberation period to review study protocols and determine their willingness to participate. All participants provided written informed consent and completed the official Chinese version of the FBS, the SSRS, the GAD-7, the PHQ-9, and the ISMI. To ensure data validity, questionnaires were completed anonymously through a dedicated research platform. The median completion duration ranged from 20–30 minutes. This study received ethical clearance from the Institutional Review Board of Chengdu Fourth Hospital (Approval No: [2022] Lun Shen Zi 71) and was prospectively registered with the Chinese Clinical Trial Registry (ChiCTR2300072081).

### 2.3 Measures

#### 2.3.1 Socio-demographic information

The socio-demographic information was collected in two domains: caregiver characteristics and corresponding adolescent profiles with NSSI. Data collected from participants included gender, age, residential status, parental status (number of dependent children), educational attainment, marital status, occupational category, monthly household income, health literacy, health insurance coverage type, and family composition. Adolescent records specifically documented the frequency of NSSI episodes during the 30-day observation period, which was determined by the clinical data recorded by psychiatrists at the latest consultation.

#### 2.3.2 Family burden scale of disease

The Family Burden Scale of Disease (FBS) (24), developed to measure the multidimensional impact of patients’ illnesses on both patients and caregivers, encompassing physical, psychological, emotional, interpersonal, and economic domains. This instrument comprises 24 items organized into six dimensions: economic burden, daily activity disruption, recreational activity limitation, family relationship strain, physical health deterioration, and psychological distress. Responses were captured using a 3-point Likert scale (0 = strongly disagree to 2 = strongly agree), with higher composite scores reflecting greater perceived family burden. Following standardized scoring procedures (total dimension score divided by number of items), scores ≥ 1 were categorized as indicating moderate-to-severe burden. The FBS has been validated and widely utilized by previous studies in the study of mental disorders (25). In the present cohort, the Chinese version of the scale demonstrated excellent internal consistency (Cronbach’s α = 0.953).

#### 2.3.3 Social support rating scale

The Social Support Rating Scale (SSRS) (26), developed to measure individuals’ perceived social support through three domains: subjective support, objective support, and support utilization. This 10-item instrument generates a composite score ranging from 12 to 66 by summating all item scores. Social support levels were classified as low (≤ 22), moderate (23–44), or high (45–66). The Chinese version has been used in a wide range of Chinese populations (27). In the present study, the scale demonstrated acceptable internal consistency (Cronbach’s α = 0.740).

#### 2.3.4 Generalized anxiety disorder-7 items

The Generalized Anxiety Disorder 7-item scale (GAD-7), developed by Spitzer (28), assesses anxiety symptoms through self-reported measures of emotional states during the preceding two-week period. This instrument contains seven items rated on a 4-point Likert scale (0 = never to 3 = nearly every day), with total scores ranging from 0 to 21. Higher total scores correspond to greater anxiety severity, with established clinical thresholds as follows: 0–4 (minimal/no anxiety), 5–9 (mild anxiety), 10–14 (moderate anxiety), and 15–21 (severe anxiety). It has shown good validity and reliability in the Chinese population (29). In the present study, the scale demonstrated excellent reliability (Cronbach’s α = 0.905).

#### 2.3.5 Patient health questionnaire-9 items

The Patient Health Questionnaire-9 (PHQ-9), developed by Kroenke (30), is a validated self-report instrument for assessing depressive symptom severity. This 9-item measure employs a 4-point Likert scale (0 = not at all to 3 = nearly every day). Higher total scores reflect greater depressive symptom severity, categorized clinically as: 0–4 (minimal depressive symptoms), 5–9 (mild depression), 10–14 (moderate depression), 15–19 (moderately severe depression), and 20–27 (severe depression). The Chinese version of the PHQ-9 has been demonstrated to be a reliable and valid measurement (29). In the current sample, the instrument demonstrated strong internal consistency (Cronbach’s α = 0.913).

#### 2.3.6 Internalized stigma of mental illness scale

The Internalized Stigma of Mental Illness Scale (ISMI), originally developed by Ritsher (31), evaluates stigma perception among individuals with mental disorders and their caregivers. This 29-item instrument assesses five core dimensions: alienation, stereotyping, discrimination, social withdrawal, and stigma resistance. Responses are recorded using a 4-point Likert scale, with higher composite scores indicating greater stigma internalization. Standardized scores (total dimension score divided by number of items per dimension) stratify stigma severity into four categories: ≤ 2 (no stigma), 2–2.5 (mild), 2.5–3 (moderate), and > 3 (severe). This scale has shown good internal consistency in the Chinese subject population (32). In the current study, the scale demonstrated excellent reliability (Cronbach’s α = 0.913).

### 2.4 Data analyses

Given the varying metric ranges of the FBS dimensions (1–6), standardized scores were computed across all six domains to facilitate the interpretation of LPA results. The LPA classification model was constructed using Mplus 8.3 software. Model selection was guided by evaluating model fit indices and clinical interpretability. Comparative fit indices included the Akaike Information Criterion (AIC), Bayesian Information Criterion (BIC), and sample size-adjusted BIC (aBIC). The AIC quantifies model fit while penalizing parameter complexity, whereas the BIC and aBIC incorporate both model parameters and sample size. As information-theoretic model selection criteria, lower values of AIC, BIC, and aBIC indicate superior model fit. Entropy values (0–1) assessed classification accuracy, with values>0.8 suggesting distinct profile separation. The Lo-Mendell-Rubin adjusted likelihood ratio test (LMRT) and bootstrapped likelihood ratio test (BLRT) statistically compared model improvements between successive class solutions, where significant p-values (*p* < 0.05) favored the k-class model over the k-1 solution. Following latent profile classification of caregiver burden severity, data were analyzed using SPSS Statistics version 27.0. Variables demonstrating statistical significance in univariate analyses (*p* < 0.05) were entered as independent predictors in a multinomial logistic regression model, the continuous numerical variables and total scale scores were used as covariates, and the results of potential profile analysis of caregiver burden served as the dependent variable. The variance inflation factor (VIF) checked potential multicollinearity between predictor variables, with VIF > 5 indicating collinearity. Statistical significance was determined at *p* < 0.05.

## 3 Results

### 3.1 Assessment of multicollinearity

To ensure no redundancy among predictors, the Variance Inflation Factor (VIF) was employed to assess multicollinearity. The results indicated that all VIF values were below 5, with the maximum value being 3.312.

### 3.2 Latent profiles determination

Fit indices for 1–5 class solutions are summarized in **Table 1**. The AIC, BIC, and aBIC values showed progressive decreases with increasing class solutions, demonstrating diminishing model improvement. The three-class solution demonstrated acceptable classification accuracy (entropy = 0.860), supported by statistically significant LMRT and BLRT results (*p* < 0.05). However, the four-class model showed non-significant LMRT improvement (*p* > 0.05). Although the four-class model showed slightly higher entropy (0.867 vs. 0.860), its fourth profile exhibited overlapping burden dimensions with clinically indistinct features, reducing practical applicability for targeted interventions. The three-class solution aligns with the clinically validated tri-level framework (low/moderate/high burden), ensuring clearer alignment with stepped-care protocols. Statistical parsimony (non-significant LMRT for four-class) further supported this choice. Thus, the three-class model balances statistical rigor and clinical translatability.

**Table 1:** Model fit indices of latent profile analysis (n = 374).

Model	AIC	BIC	aBIC	Entropy	*P*-value		Latent class probability
					LMR	BLRT	
1 class	3872.612	3919.703	3881.631	—	—	—	—
2 class	3116.527	3191.088	3130.807	0.866	<0.001	<0.001	0.516/0.484
3 class	2972.683	3074.714	2992.223	0.860	0.003	<0.001	0.345/0.366/0.289
4 class	2837.105	2966.605	2861.905	0.867	0.068	<0.001	0.238/0.131/0.348/0.283
5 class	2747.551	2904.521	2777.612	0.866	0.589	<0.001	0.107/0.227/0.318/0.128/0.219

AIC, Akaike Information Criterion; BIC, Bayesian Information Criterion; aBIC, Sample size-adjusted BIC; LMR, Lo-Mendell-Rubin adjusted likelihood ratio test; BLRT, bootstrapped likelihood ratio test.

**Figure 1:**
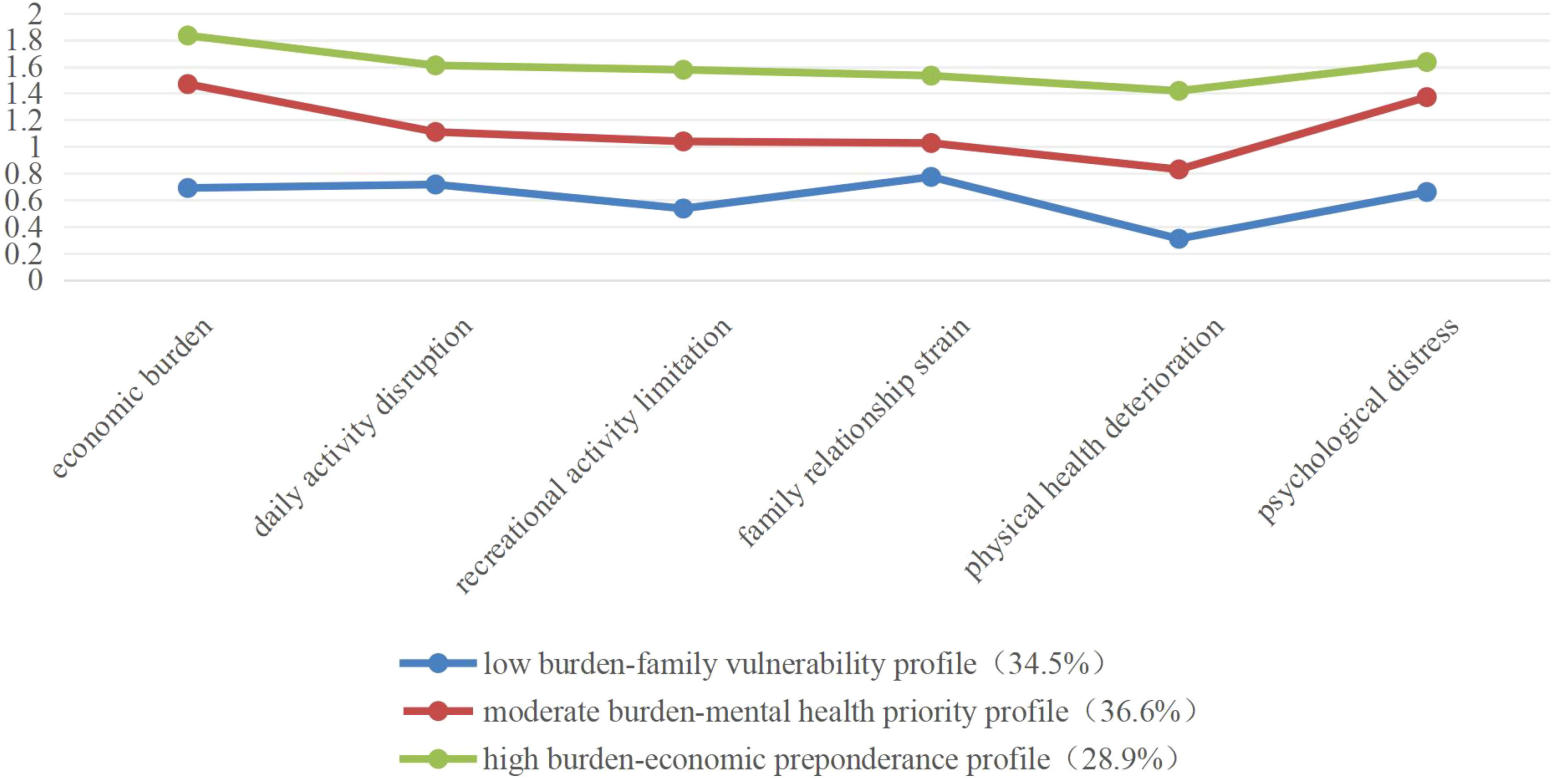
Profiles of latent classes of caregiver burden.

**Figure 1** illustrates the three distinct caregiver burden profiles identified via LPA across six measurement domains. Class 1 accounted for 34.5% of the sample, followed by Class 2 (36.6%) and Class 3 (28.9%).

### 3.3 Socio-demographic characteristics of the participants

Analysis of 374 completed questionnaires revealed a caregiver cohort predominantly female (63.6%, n = 238), with 92.2% aged ≥ 35 years. The majority reported multiparous status (58.6% with ≥ 2 children) and urban residency (53.5%). Twelve variables, including demographic characteristics (gender, residential status, parental status, educational attainment, monthly household income, health literacy, family composition, and the frequency of NSSI episodes) and mental health indicators (social support, anxiety, depression, and internalized stigma), showed statistically significant differences among the three caregiver burden classes (*p < *0.05), while other variables showed no statistical differences (*p* > 0.05), as shown in **Table 2**.

**Table 2:** Comparison of clinical and socio-demographic across three profiles (n = 374).

Variables		Low burden-family vulnerability profile (n = 128)	Moderate burden-mental health priority profile (n = 109)	High burden-economic preponderance profile (n = 137)	χ2/F	*P*
Gender	Female	62(48.4%)	93(85.3%)	83(60.6%)	35.479	<0.001
	Male	66(51.6%)	16(14.7%)	54(39.4%)		
Age	≤ 35	10(7.8%)	7(6.4%)	12(8.8%)	2.123	0.713
	36 ~ 45	65(50.8%)	61(56.0%)	79(57.6%)		
	≥ 46	53(41.4%)	41(37.6%)	46(33.6%)		
Residential status	Rural	11(8.6%)	41(37.6%)	30(21.9%)	28.964	<0.001
	City	117(91.4%)	68(62.4%)	107(78.1%)		
Parental status	Own only one child	68(53.1%)	36(33.0%)	51(37.2%)	11.383	0.003
	Own two or more child	60(46.9%)	73(67.0%)	86(62.8%)		
Education attainment	Junior high school and below	51(39.8%)	86(78.9%)	76(55.5%)	36.818	<0.001
	High school and above	77(66.2%)	23(21.1%)	61(44.5%)		
Marital status	Married	105(82.0%)	82(75.2%)	111(81.0%)	1.923	0.382
	Remarried/Divorced/widowed	23(18.0%)	27(24.8%)	26(19.0%)		
Occupation category	On duty	112(87.5%)	91(83.5%)	114(83.2%)	1.135	0.567
	Off duty	16(12.5%)	18(16.5%)	23(16.8%)		
Monthly family income (CNY)	< 5000	37(28.9%)	75(68.8%)	55(40.1%)	48.325	<0.001
	5001 ~ 10000	41(32.0%)	27(24.8%)	43(31.4%)		
	> 10000	50(39.1%)	7(6.4%)	39(28.5%)		
Health literacy	Little understanding	84(65.6%)	95(87.2%)	95(69.3%)	15.627	<0.001
	Enough understanding	44(34.4%)	14(12.8%)	42(30.7%)		
Health insurance coverage type	Self - funded	25(19.5%)	14(12.8%)	20(14.6%)	2.683	0.612
	Provincial medical insurance	36(28.1%)	30(27.5%)	42(30.7%)		
	Urban medical insurance	67(52.4%)	65(59.7%)	75(54.7%)		
Family composition	Nuclear family	77(60.1%)	45(41.3%)	73(53.3%)	15.706	0.015
	Stem family	27(21.1%)	38(34.9%)	40(29.2%)		
	Single-parent family	12(9.4%)	21(19.3%)	18(13.1%)		
	Other families	12(9.4%)	5(4.5%)	6(4.4%)		
The frequency of NSSI episodes	≤ 5 times/month	83(64.8%)	38(34.9%)	68(49.6%)	21.239	<0.001
	> 5 times/month	45(35.2%)	71(65.1%)	69(50.4%)		
Social support		42.28 ± 7.52	31.65 ± 6.55	37.94 ± 7.43	64.158	<0.001
Anxiety		4.48 ± 3.82	11.14 ± 4.05	7.42 ± 3.92	84.867	<0.001
Depression		4.10 ± 4.12	11.71 ± 4.79	7.74 ± 4.71	82.459	<0.001
Internal Disease Shame		49.06 ± 9.13	63.46 ± 9.05	56.51 ± 9.50	71.714	<0.001

CNY, Chinese Yuan; Health literacy, basic understanding of NSSI; Nuclear family, a household comprising two generations (parents and dependent children) with no other kin residing together; Stem family, a multigenerational household comprising parents, one married child with their spouse and children. Single-parent family, a household headed by one parent raising unmarried children without a cohabiting partner.

Profile nomenclature was derived from standardized scoring patterns across classes: Class 1 (n = 128) showed uniformly low domain scores except elevated family relationship strain, designated as “low burden-family vulnerability profile”. Class 2 (n = 109) presented a moderate overall burden with prominent psychological distress scores, labeled “moderate burden-mental health priority profile”. Class 3 (n = 137) exhibited clinically elevated burdens across all domains, particularly economic strain, categorized as “high burden-economic predominance profile”.

### 3.4 Predictor of latent profile membership

Using the profiles of latent classes of caregiver burden as the dependent variable, the low burden-family vulnerability profile as the reference, and the statistically significant factors in the univariate analysis as the independent variables. Multiple logistic regression analyses (**Table 3**) revealed significant associations between caregiver burden profiles and both demographic characteristics (gender, residential status) and mental health indicators (social support, anxiety, depression, internal disease shame) (all *p* < 0.05). Female caregivers, rural residents, diminished social support, and caregivers with higher anxiety were more likely to belong to the moderate burden profile. Heightened depression symptoms and stronger illness-related stigma were more likely to belong to both the moderate burden-mental health priority profile and the high burden-economic preponderance profile. Additionally, the stress-buffering hypothesis (33) posits that social support attenuates the psychological impacts of chronic stressors. It was hypothesized that caregivers’ social support would modify the association between adolescent NSSI frequency and caregiver burden. The NSSI frequency _*_ social support interaction was tested across latent classes, the result showed no significant effects in either the moderate burden-mental health priority profile or the high burden-economic preponderance profile (*p*>0.05).

**Table 3:** The multifactor analysis of logistic regression.

Variable		Moderate burden-mental Health priority profile				High burden-economic preponderance profile			
		*β*	*p*	OR	95% CI	*β*	*p*	OR	95% CI
Gender	Female	1.324	0.005	3.760	(1.484,9.529)	0.173	0.583	1.189	(0.640,2.209)
Residential status	Rural	1.299	0.027	3.666	(1.162,11.563)	0.755	0.130	2.127	(0.800,5.653)
Parental status	Own only one child	0.587	0.201	1.798	(0.732,4.418)	-0.390	0.227	0.677	(0.360,1.274)
Education attainment	Junior high school and below	-0.157	0.749	0.855	(0.328,2.231)	-0.223	0.528	0.800	(0.401,1.598)
Marital status	< 5000	0.612	0.361	1.844	(0.496, 6.853)	0.133	0.763	1.142	(0.482,2.705)
	5001 ~ 10000	0.015	0.981	1.015	(0.293,3.510)	-0.259	0.506	0.772	(0.359,1.658)
Health literacy	Little understanding	0.892	0.076	2.440	(0.912,6.532)	0.072	0.832	1.075	(0.552,2.095)
Family composition	Nuclear family	0.932	0.241	2.540	(0.535,12.062)	0.916	0.173	2.498	(0.670, 9.320)
	Stem family	1.549	0.070	4.709	(0.884,24.095)	1.608	0.055	5.174	(0.977, 27.448)
	Single-parent family	1.129	0.215	3.092	(0.519,18.427)	1.100	0.152	3.005	(0.668, 13.519)
The frequency of NSSI episodes	>5 times/month	-1.714	0.463	0.180	(0.002, 17.501)	0.576	0.775	1.778	(0.048, 65.692)
Social support		-0.124	0.016	0.884	(0.799,0.977)	-0.017	0.637	0.983	(0.914, 1.056)
Anxiety		0.168	0.027	1.183	(1.019,1.373)	0.056	0.377	1.058	(0.934, 1.199)
Depression		0.175	0.010	1.191	(1.043,1.361)	0.122	0.037	1.130	(1.007, 1.268)
Internal disease shame		0.097	<0.001	1.102	(1.051,1.157)	0.061	0.001	1.063	(1.025, 1.103)
Interaction effects	The frequency of NSSI episodes _*_ Social support	-0.005	0.932	0.995	(0.881, 1.123)	-0.044	0.324	0.957	(0.878, 1.044)

Reference group is low burden-family vulnerability profile; OR, Odds ratio; 95% Cl, 95% Confidence Interval.

* indicates the interaction term

## 4 Discussion

LPA identified three distinct burden profiles among caregivers of adolescents with NSSI: low burden-family vulnerability profile (34.5%), moderate burden-mental health priority profile (36.6%), and high burden-economic preponderance profile (28.9%). This typology demonstrates substantial heterogeneity in caregiver burden manifestation, with 65.5% of caregivers of adolescents with NSSI experiencing moderate-to-severe burden levels, significantly exceeding the 38.2% prevalence observed in schizophrenia caregiver populations (34). The elevated burden may reflect the developmental specificities of adolescent care recipients, who concurrently face academic pressures and extended treatment durations (35).

This study revealed that female caregivers were more likely to belong to the moderate burden-mental health priority profile, consistent with existing evidence demonstrating that mothers of adolescents with NSSI report elevated depression, anxiety, and stress scores (36). Gender-based differences in personality traits may contribute to this pattern, as women generally demonstrate heightened emotional sensitivity and greater susceptibility to irritability compared to men (37). Furthermore, research indicates that female relational strengths foster stronger therapeutic alliances with adolescents, enhancing empathy-driven responses in NSSI care (37). These intensified emotional connections may predispose female caregivers to elevated anxiety and depressive symptoms secondary to caregiving stressors, thereby exacerbating familial burden (38). However, traditional Confucian values in China, which emphasize a gendered division of labor conceptualized as men dominate outside, and women dominate inside, continue to shape the distribution of economic pressures (39). Consequently, female caregivers are underrepresented in high burden-economic preponderance profile. These findings underscore the necessity for gender-specific psychological support interventions, particularly targeted emotion regulation training for female caregivers to mitigate negative affective states and optimize caregiving outcomes.

This study demonstrated that rural caregivers were more likely to belong to the moderate burden-mental health priority profile, aligning with findings reported by Siddiqui (40). This disparity may stem from systemic healthcare challenges in rural settings, including resource scarcity, underdeveloped healthcare infrastructure, and insufficient primary care personnel (41). Additionally, rural caregivers generally demonstrate lower educational attainment, constraining their capacity to obtain condition-specific knowledge and access evidence-based interventions (42). Heightened NSSI-related stigmatization in these populations may further predispose caregivers to anxiety, depression, and comorbid psychological distress (43). However, emerging evidence shows that China’s Rural Cooperative Medical Insurance system reduces out-of-pocket medical costs by 40%, effectively lowering caregivers’ financial stress (44). Consequently, rural caregivers are underrepresented in high burden-economic preponderance profile. These findings highlight the need for multisectoral approaches to improve early case detection and care. Coordinated implementation could reduce caregiver distress through timely adolescent mental health services and family support programs.

Social support was more likely to belong to the moderate burden-mental health priority profile. The social buffering theory (33) asserts that perceived social support mitigates stress responses by enhancing individuals’ adaptive capacities to societal challenges. From a family systems perspective, externally derived social support attenuates the disruptive effects of stressors on family dynamics. Robust social networks not only foster psychological resilience and somatic health maintenance but also reinforce self-efficacy and goal-directed behaviors through positive cognitive reinforcement (45). Consequently, implementing multidimensional support systems proactively reduces caregiver burden (46). Empowering caregivers to mobilize community resources, engage in peer support networks, and adopt help-seeking behaviors is critical (47). Concurrently, systemic integration of caregiver support into national healthcare frameworks further ensures sustainable burden alleviation through institutionalized assistance mechanisms.

This study revealed that elevated anxiety scores were more likely to belong to the moderate burden-mental health priority profile. Emerging neurobehavioral evidence links NSSI to addiction-like neuroplasticity, characterized by chronic engagement-induced dysregulation of hippocampal-amygdala circuits in adolescents, underlying both heightened suicide risk and caregiver distress potentiation (48). Mechanistically, recurrent NSSI episodes create cyclical caregiving crises through progressive symptom escalation requiring intensified monitoring and the emergence of treatment-resistant behavioral patterns (49). These clinical trajectories synergistically compound caregiver anxiety through anticipatory vigilance and resource depletion (50). This pathophysiology underscores the imperative for Multidisciplinary care models integrating trauma-informed psychotherapy with caregiver psychoeducation (51).

This study demonstrated that elevated caregiver depression scores were more likely to be both the moderate burden-mental health priority profile and the high burden-economic preponderance profile. Caregivers’ persistent concerns about NSSI’s impact on adolescent development trajectories, particularly regarding academic performance, vocational prospects, and interpersonal relationships, were found to progressively induce physical exhaustion, emotional distress, and impaired social functioning (45). Chronic exposure to these biopsychosocial stressors established cyclical patterns of depressive symptomatology, which amplified familial disease burden through psychosomatic mechanisms (10). The clinical complexity of NSSI when comorbid with psychiatric disorders (e.g., major depressive disorder, borderline personality disorder) necessitates multimodal interventions, particularly psychotherapy as a core therapeutic component (52). Within China’s healthcare system, the exclusion of psychotherapeutic services from national health insurance coverage creates substantial financial burdens for families through out-of-pocket payments (53). This economic strain is intensified when caregivers require extended leave or employment termination to accommodate treatment schedules, generating compounded financial and psychological consequences. These findings highlight the necessity for implementing standardized caregiver mental health screening protocols and evidence-based support systems.

This study demonstrated that the elevated caregiver internalized stigma levels were more likely to be categorized in both the moderate burden-mental health priority profile and the high burden-economic preponderance profile. Caregivers of adolescents with NSSI frequently develop chronic self-blame and shame due to recurrent symptom relapse cycles and pervasive societal stigma (54). This internalized stigma initiates a self-perpetuating cycle of social withdrawal, progressively eroding social support networks through avoidance behaviors (11). Stigmatized caregivers often demonstrate a pathological denial of adolescent psychopathology, actively resisting professional interventions to avoid perceived social judgment. Such maladaptive coping perpetuates unresolved distress and inadvertently reinforces NSSI behaviors via emotional contagion mechanisms (46). Multisector interventions should integrate psychoeducational programs targeting NSSI neurobiological mechanisms and psychosocial risk factors. Furthermore, empirical evidence demonstrates that caregivers derive substantial benefits from structured support networks, including family, friends, and peer support. Evidence from randomized trials indicates that structured peer support networks reduce internalized stigma and enhance adaptive coping strategies (55). Qualitative data further elucidate how caregiver peer alliances facilitate practical strategy exchange and cognitive restructuring of caregiving challenges (56), while fostering empowerment through collective resilience-building (57, 58).

Cultural contexts profoundly influence caregiver burden dynamics in adolescents with NSSI. In collectivist societies such as China, multigenerational households magnify caregiving responsibilities through filial piety traditions, while simultaneously exacerbating family shame associated with mental health stigma (59). Under patriarchal norms prevalent in China, female caregivers disproportionately internalize self-blame for NSSI through attribution to familial inadequacy, thereby compounding psychological distress (60).

## 5 Limitations

While this investigation provides notable insights, three constraints warrant acknowledgment. Primarily, the cross-sectional design precludes causal inferences regarding observed associations, and reliance on self-reported data introduces potential biases. Future studies could employ longitudinal designs with objective measures to delineate comprehensive burden trajectories. Secondly, the exclusive focus on caregivers within Sichuan Province raises concerns about geographical generalizability. Future studies should conduct multi-regional sampling across diverse geographical settings to validate findings beyond Sichuan Province. Furthermore, participant recruitment was restricted to caregivers of hospitalized adolescents, thereby potentially overlooking critical burden dimensions in community-dwelling caregivers who have not accessed clinical services. Future studies should expand recruitment to include non-clinical community-based caregivers of adolescents to capture underrepresented burden profiles.

## 6 Conclusion

This study demonstrates the heterogeneity of caregiver burden among adolescents with NSSI through LPA, identifying three distinct profiles: low burden-family vulnerability, moderate burden-mental health priority, and high burden-economic preponderance. These findings underscore the need for profile-specific interventions to address multidimensional burden drivers. Mental health professionals are encouraged to prioritize anxiety management and stigma reduction initiatives tailored for families. Policymakers may explore rural-focused partnerships while embedding caregiver psychosocial evaluations into NSSI care protocols. Caregivers may benefit from accessing digital peer-support platforms through real-time resource sharing. Future research should translate these findings into precision care models that address both psychological distress and structural disparities.

## Data Availability

The raw data supporting the conclusions of this article will be made available by the authors, without undue reservation.
